# Prognostic and immunological significance of metastasis associated lung adenocarcinoma transcript 1 among different kinds of cancers

**DOI:** 10.1080/21655979.2021.1955511

**Published:** 2021-07-24

**Authors:** Lili Guo, Xiuwen Zhang, Hongli Pan, Yang Li, Jing Wang, Lin Li, Yafang Dong, Xinxin Du, Jun Chen, Fengjie Guo

**Affiliations:** a Tianjin Key Laboratory of Lung Cancer Metastasis and Tumor Microenvironment, Tianjin Lung Cancer Institute, Tianjin Medical University General Hospital, Tianjin, People’s Republic of China; b Precision Medicine Center, The Affiliated People’s Hospital of Shanxi Medical University, Taiyuan, People’s Republic of China; c School of Medicine, South China University of Technology, Guangzhou, People’s Republic of China

**Keywords:** MALAT1, prognosis, immune cell infiltrating, cancer

## Abstract

LncRNAs belong to the type of noncoding RNA transcripts, which exceed 200 nucleotides in size. MALAT1 as one of the earlier identified lncRNAs in cancer is investigated by more and more scientific researchers. Expression, clinical significance and function of MALAT1 in pan-cancer exist as big difference. To detect the expression and clinical significance of MALAT1 gene precisely and comprehensively among different kinds of cancers, some classical databases such as GEPIA, TIMER, KM Plotter, and PrognoScan were fully applied. An immunological role of MALAT1 among different kinds of cancers was also determined in TIMER database. Our results showed that MALAT1 was differently expressed in different kinds of cancers using GEPIA, Oncomine, and TIMER databases to analyze. Especially, MALAT1 high RNA level was related to the early stage in lung and gastric cancer patients. MALAT1 expression was closely related to prognosis among different cancer patients. Furthermore, expression of MALAT1 was related to tumor immune cell infiltrating. Expression level of MALAT1 was also related to immune makers such as macrophage, T cell, NK cells, and so on. These findings indicate that MALAT1 could be a potential prognostic biomarker of some kinds of cancer and was significantly correlated with tumor-infiltrating immune cells in a wide variety of cancers.

## GRAPHICAL ABSTRACT

**Figure:**
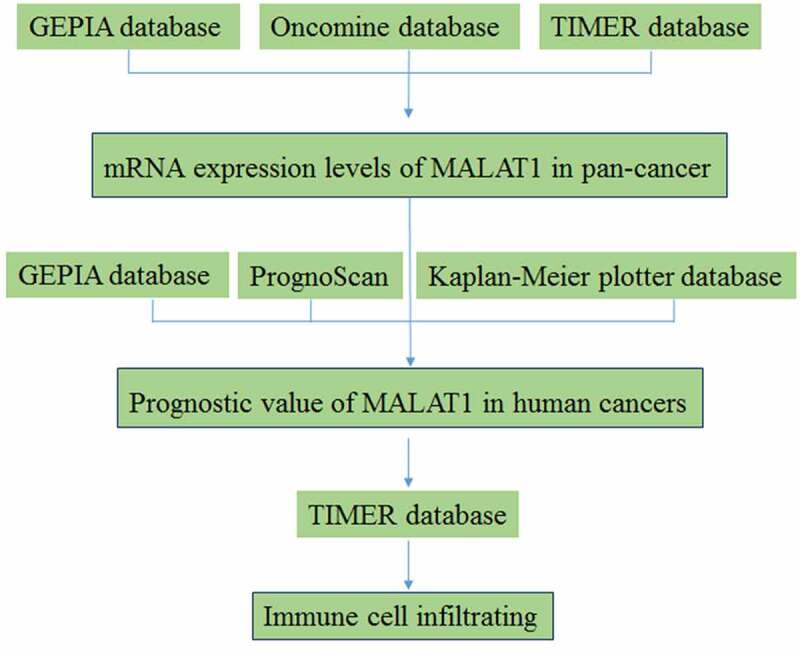

## Introduction

The length of long noncoding RNAs exceeds 200 nucleotides. Given the nonexistence of protein coding region, lncRNAs lose the ability of encoding proteins. LncRNAs have diverse functions in the pathogenesis and development of tumors, including drug resistance and cell proliferation [1–3]. However, the complete mechanisms of action of some lncRNAs are still less. As an important functional lncRNA, MALAT1 has attracted our attention.

MALAT1, with length more than 8,000 nts, is also one abundant and conserved long noncoding RNA in mammalian species [4–6]. MALAT1 is widely expressed in human tissues and regulates its growth and development [7]. At the beginning, MALAT1 was found to be a poor prognostic marker for cancer patients [8]. The accumulating evidence demonstrated the role of MALAT1 as an oncogene in multiple cancers [9,10]. However, different or opposite results also were raised in some studies: tumor suppressive function of MALAT1 [11,12]. For the mouse, after MALAT1 was knocked out, the normal growth was not affected [13]. Therefore, the possible biological functions and mechanisms of MALAT1 in tumor progression were not still clear.

The treatment of cancer remains a challenge, and incidence rate or mortality of cancer is high [14]. Immunotherapy as a kind of dated and fresh way to treat cancer represents a new trend of cancer treatment and is growing well received [15]. The key discoveries in cancer immunotherapy such as the killing effects of CTLA-4 and programmed death-ligand 1/programmed death-1 (PD-L1/PD-1) inhibition make the immunotherapy more attractive [16].

The aim of our study is to explore the role of MALAT1 in different types of cancers. Therefore, in this study, the data in some public databases were used to comprehensively study the expression and prognostic significance of MALAT1 in cancers. The immunological characteristics of MALAT1 in different cancers were also determined in TIMER database. Our results allow us to better understand the different roles of MALAT1 in different cancers.

## Materials and methods

### GEPIA database analysis

GEPIA is an online tool to determine the RNA data obtained from TCGA and GTEx database [17]. GEPIA is utilized to study gene expression in tumor/normal samples, gene correlation, and survival.

### Oncomine database analysis

MALAT1 expression in multiple tumors was also detected by Oncomine website. The Oncomine Platform as a cancer microarray database was built by physicians, scientists, and software engineers at the University of Michigan to mine cancer data based on web service (https://www.oncomine.org/) [18].

### TIMER database analysis

TIMER as a database of analyzing immune infiltrates included 10,009 samples among more than 20 cancer kinds from TCGA [19,20]. Gene expressions and survival were also displayed using different forms in TIMER database. We analyzed MALAT1 RNA level in multiple cancers and the correlation of MALAT1 RNA level with the immune cell infiltrating.

### Survival Analysis in GEPIA, Kaplan–Meier Plotter, and PrognoScan

The survival in multiple cancers was detected in Kaplan–Meier Plotter [21], GEPIA, and PrognoScan [22].

### Statistical analysis

Gene expression from GEPIA, Oncomine, and TIMER is shown with fold change and p-value. To generate survival curves, the log-rank test was applied in GEPIA, PrognoScan, and KM Plotter. The correlation analysis was conducted using Spearman’s correlation. A student’s t-test was used for gene expression analyses between two groups; one-way ANOVA test was used when comparing multiple groups. A p < 0.05 has a statistical significance.

## Results

Expression, clinical significance, and function of lncRNA MALAT1 in pan-cancer exist as big difference. To detect the expression and clinical significance of MALAT1 gene better, precisely and comprehensively among different kinds of cancers, some classical databases such as GEPIA, TIMER, KM Plotter, and PrognoScan were fully applied. Our results showed that MALAT1 was differently expressed in different kinds of cancers. MALAT1 expression was closely related to prognosis and tumor immune cell infiltrating among different cancer patients.

### mRNA expression levels of MALAT1 in pan-cancer

**Figure 1.**
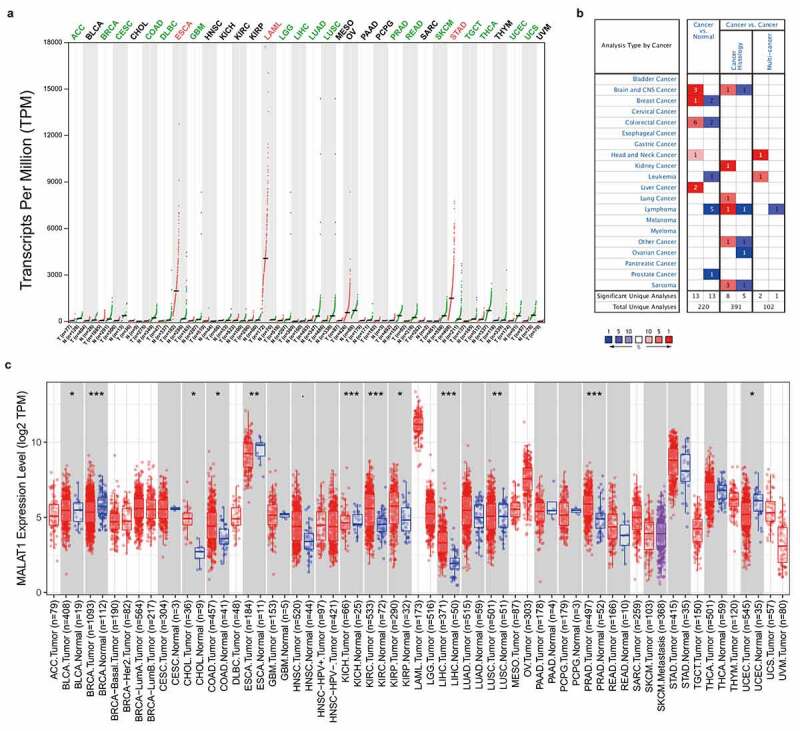
**mRNA expression levels of MALAT1 in pan-cancer**. (a) The expression level of MALAT1 in tumor and normal tissues was analyzed by GEPIA database. (b) The expression level of MALAT1 in Oncomine database. (c) TIMER database was used to investigate the mRNA expression of MALAT1 in tumor and normal tissues

To evaluate the RNA level of MALAT1 across various cancers, MALAT1 level was first analyzed by GEPIA. This platform enrolled 9,736 tumors and 8,587 normal samples from the TCGA and the GTEx projects. The results illustrated that the RNA level of MALAT1 in ESCA (esophageal cancer), LAML (acute myeloid leukemia), and STAD (stomach adenocarcinoma) was higher than normal tissues. And a low expression of MALAT1 was widely found in ACC (adrenocortical carcinoma), BRCA (breast invasive carcinoma), CESC (cervical squamous cell carcinoma and endocervical adenocarcinoma), COAD (colon adenocarcinoma), DLBC (diffuse large B-cell lymphoma), GBM (glioblastoma multiforme), LGG (brain lower grade glioma), LIHC (liver hepatocellular carcinoma), LUAD (lung adenocarcinoma), LUSC (lung squamous cell carcinoma), PRAD (prostate adenocarcinoma), READ (rectum adenocarcinoma), SKCM (skin cutaneous melanoma), TGCT (testicular germ cell tumors), THCA (thyroid carcinoma), UCEC (uterine corpus endometrial carcinoma), and UCS (uterine carcinosarcoma) compared with normal tissues (Figure 1(a)).

Next, MALAT1 level was evaluated by oncomine database. This platform involved 715 datasets and 86,733 samples. MALAT1 level in colorectal, brain and CNS, breast, liver and head and neck cancer tissues was upregulated compared to normal tissues, whereas low MALAT1 level in breast, colorectal and prostate cancers, leukemia and lymphoma compared with normal tissues was revealed in some data sets (Figure 1(b).

Finally, TIMER was utilized to analyze MALAT1 RNA level. MALAT1 RNA level was high in CHOL (cholangiocarcinoma), COAD (colon adenocarcinoma), KICH (kidney chromophobe), KIRC (kidney renal clear cell carcinoma), LIHC (liver hepatocellular carcinoma), LUSC (Lung squamous cell carcinoma), PRAD (prostate adenocarcinoma), whereas MALAT1 expression was low in BLCA (bladder urothelial carcinoma), BRCA (breast invasive carcinoma), ESCA (esophageal carcinoma), and UCEC (uterine corpus endometrial carcinoma; Figure 1(c)).

### Prognostic value of MALAT1 in human cancers

**Figure 2.**
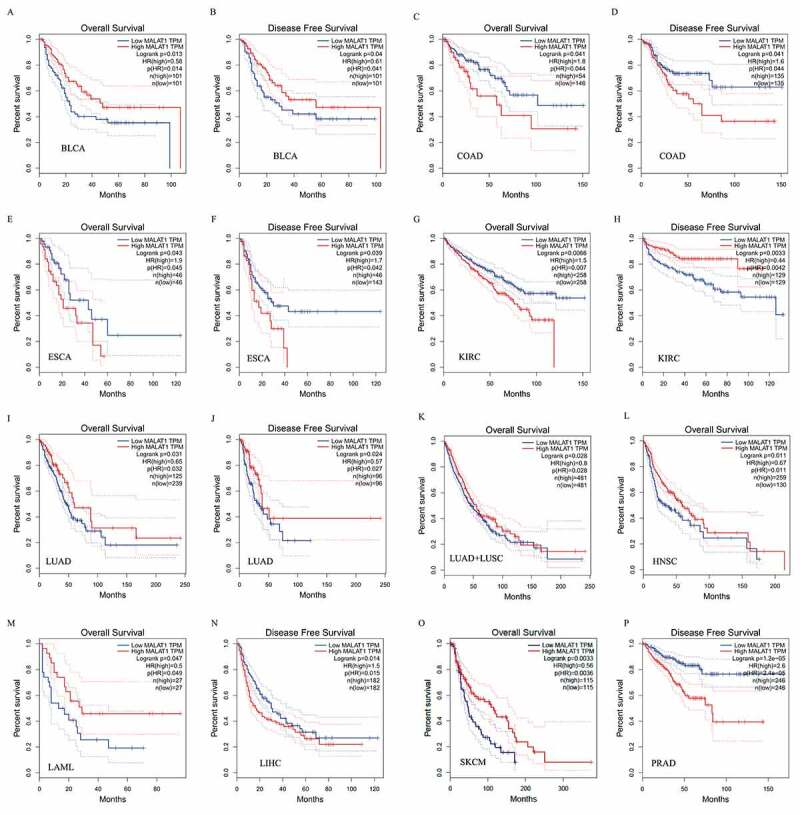
**Survival curves of MALAT1 expression in different tumors from the GEPIA database**. MALAT1 high expression was associated with a worse prognosis in COAD (c and d), ESCA (e and f), LIHC (n), KIRC (h) and PRAD (p). Meanwhile, MALAT1 high expression was associated with a better prognosis in BLCA (a and b), LUAD (i and j)/LUAD+LUSC (k), HNSC (l), KIRC (g), SKCM (o), and LAML (m)

The survival analysis of MALAT1 expression in different cancer patients was evaluated by GEPIA database. The results illustrated that MALAT1 RNA level was related to 11 types of cancers, including BLCA, COAD,LAML, LIHC, ESCA, KIRC, LUAD, HNSC, KIRC, SKCM, and PRAD (Figure 2). Among them, upregulaton of MALAT1 level correlated with a poor prognosis in COAD (OS HR = 1.8, p = 0.041; DFS HR = 1.6 p = 0.041), ESCA (OS HR = 1.9, p = 0.043; DFS HR = 1.7 p = 0.039), LIHC (DFS HR = 1.5 p = 0.014), KIRC (OS HR = 1.5, p = 0.0066), and PRAD (DFS HR = 2.6 p < 0.0001). Reversely, MALAT1 high level was related to a better prognosis in BLCA (OS HR = 0.58, p = 0.013; DFS HR = 0.61 p = 0.04), LUAD (OS HR = 0.65, p = 0.031; DFS HR = 0.57 p = 0.024) /LUAD+LUSC (OS HR = 0.8, p = 0.028), HNSC (OS HR = 0.67, p = 0.011), SKCM (OS HR = 0.56, p = 0.003), and LAML (OS HR = 0.5, p = 0.047). Interestingly, MALAT1 played the two-faced role in KIRC: a negative prognostic factor in OS (HR = 1.5, p = 0.0066) and a positive prognostic factor in DFS (HR = 0.44, p = 0.0033).

In addition, the PrognoScan database was also used to study prognostic significance of MALAT1 in human cancers (Supplementary Figure 1).

### Effect of high expression of MALAT1 on gastric and lung cancer progression in early stage

Because of the MALAT1 high expression in gastric cancer patients, we next explored the relationship between MALAT1 RNA level and clinicopathological features using KM plotter databases to understand the underlying mechanisms. As a result, we found that MALAT1 high expression was related to poor OS and FP (First Progression) in stage, stage T, stage N, differentiation, and so on (Table 1). Interestingly, MALAT1 high level correlated with stage 1 (OS HR = 0.27, P = 0.011; FP HR = 0.3 P = 0.0239) and stage 3 (OS HR = 0.54, P = 0.0012; FP HR = 0.61 P = 0.0158) of gastric cancer patients but not stage 2 and stage 4 (except for OS; Table 1). MALAT1 high RNA level was also related to stage T3, stage N0, N1, N2, N1 + 2 + 3, stage M0, but not stage 4 (except for OS), stage N3, and stage M (Table 1). Moreover, MALAT1 high expression was related both to Lauren classification (intestinal and diffuse) and differentiation (poor and moderate) regardless of whatever levels. Especially, of the clinical parameters, we noticed that MALAT1 high level was related to early stage in gastric cancer patients but not late stage. These suggested that MALAT1 expression has the potential to impact gastric cancer patient prognosis in early stage.

**Table 1.** Correlation of MALAT1 expression and clinical prognosis in gastric cancer by Kaplan–Meier plotter

Clinicopathological characteristics	Overall survival			First progression		
	n	Hazard ratio	*P*-value	n	Hazard ratio	*P*-value
Sex						
Female	187	0.52 (0.32–0.84)	0.0063	179	0.48 (0.27–0.83)	0.0075
Male	349	0.57 (0.42–0.77)	0.0002	341	0.67 (0.5–0.89)	0.0064
Stage						
1	62	0.27 (0.09–0.8)	0.011	60	0.3 (0.1–0.91)	0.0239
2	135	0.44 (0.18–1.06)	0.0599	131	0.49 (0.22–1.1)	0.0775
3	197	0.54 (0.37–0.79)	0.0012	186	0.61 (0.41–0.92)	0.0158
4	140	0.58 (0.39–0.86)	0.0069	141	0.71 (0.48–1.04)	0.0802
Stage T						
2	241	0.67 (0.44–1.02)	0.0597	239	0.61 (0.37–1.01)	0.0524
3	204	0.51 (0.35–0.75)	0.0005	204	0.63 (0.44–0.91)	0.0123
4	38	1.73 (0.75–4.01)	0.1934	39	0.63 (0.29–1.38)	0.2448
Stage N						
0	74	0.09 (0.01–0.64)	0.0024	72	0.09 (0.01–0.71)	0.0043
1	225	0.48 (0.32–0.73)	0.0005	222	0.48 (0.33–0.71)	0.0002
2	121	0.44 (0.27–0.72)	0.0007	125	0.46 (0.29–0.75)	0.0011
3	76	0.55 (0.32–0.94)	0.0262	76	0.64 (0.38–1.1)	0.1017
1 + 2 + 3	422	0.57 (0.44–0.74)	2.6e-5	423	0.61 (0.47–0.8)	0.0002
Stage M						
0	444	0.63 (0.48–0.84)	0.0014	443	0.63 (0.45–0.89)	0.0079
1	56	0.33 (0.16–0.66)	0.001	56	0.58 (0.29–1.16)	0.1174
Lauren classification						
Intestinal	269	0.66 (0.45–0.96)	0.0286	263	0.7 (0.49–1.01)	0.0539
Diffuse	240	0.48 (0.34–0.68)	2.0e-5	231	0.54 (0.38–0.76)	0.0004
Differentiation						
Poor	121	1.82 (1.12–2.97)	0.0146	121	1.67 (1.06–2.65)	0.0267
Moderate	67	3.06 (1.17–8.02)	0.0174	67	2.64 (1.09–6.42)	0.0265

In addition, the association of MALAT1 RNA level with prognosis of lung cancer was also analyzed using Kaplan–Meier plotter database. High mRNA level of MALAT1 was related to better OS or FP of lung cancer patients in sex, stage, and chemotherapy (Supplementary Table 1). Especially, MALAT1 high RNA level was related to adenocarcinoma, stage 1 and stage 2 (except for OS), stage T1, but not stage 3 or stage T2, stage 3, and stage 4. These suggested that MALAT1 expression has also influenced lung cancer patient prognosis in early stage.

### MALAT1 expression was associated with immune cell infiltrating in lung and bladder cancer

**Figure 3.**
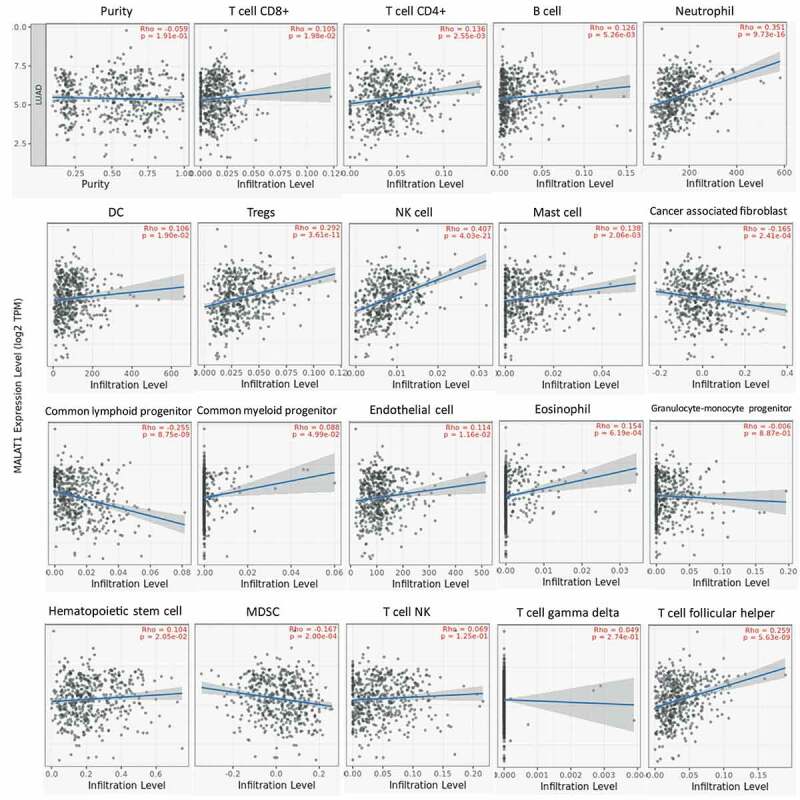
**MALAT1 expression was associated with immune cell infiltrating in lung cancer**. In LUAD, MALAT1 expression was related to CD8 + T-cell, CD4 + - cell, B cell, neutrophil cell, DC cell, Tregs cell, NK cell, Mast cell, cancer-associated fibroblast, common lymphoid progenitor, common myeloid progenitor, endothelial cell, eosinophil, hematopoietic stem cell, MDSC, and T-cell follicular helper cell infiltration

The lymphocytes infiltrating in tumors were considered to link the prognosis and response to tumor therapy [23,24]. So, we also studied the association of MALAT1 level with immune cell infiltrating in tumor with TIMER website. Initially, we assessed the tumor purity and found that RNA level of MALAT1 was significantly related to the tumor purity in eight types of tumors (Supplementary figure 2). In addition, there was an association of MALAT1 mRNA level with infiltrating immune cell in various tumors. In LUAD, MALAT1 mRNA level was related to CD8 + T cell, CD4 + T cell, B cell, neutrophil cell, DC cell, Tregs cell, NK cell, Mast cell, cancer-associated fibroblast, common lymphoid progenitor, common myeloid progenitor, endothelial cell, eosinophil, hematopoietic stem cell, MDSC, and T-cell follicular helper cell infiltration (Figure 3). In BLCA, MALAT1 RNA level correlated with CD8 + T cell, CD4 + T cell, DC cell, Tregs cell, Mast cell, cancer-associated fibroblast, common lymphoid progenitor, eosinophil, MDSC, T cell NK, and T cell follicular helper cell infiltration (Supplementary figure 3). In LUAD and BLCA, MALAT1 were both associated with CD8 + T cell, CD4 + T cell, DC cell, Tregs cell, Mast cell, cancer-associated fibroblast, common lymphoid progenitor, eosinophil, MDSC, and T-cell follicular helper cell infiltration. These indicated the crucial role of MALAT1 in immune cell infiltrating in tumors.

### The correlation of MALAT1 expression with macrophage

**Figure 4.**
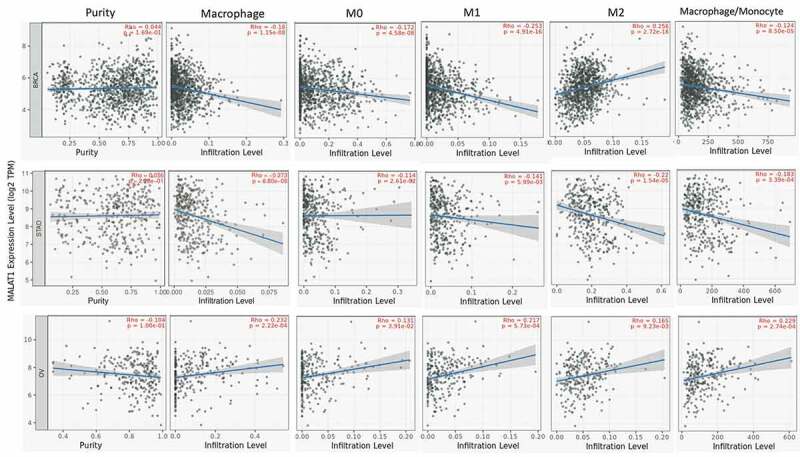
**The correlation of MALAT1 expression with macrophage**. MALAT1 expression was significantly correlated with macrophage in multiple tumors including BRCA, STAD, and OV

Macrophage as an important cell in the innate immune system diversely functions in normal and disease development [25,26]. In this analysis, we found that MALAT1 expression was related to macrophage in multiple tumors (26 in 40 types of tumors). Especially, MALAT1 mRNA level was almost negatively related to macrophage, M0, M1, M2, and macrophage/monocyte in BRCA and STAD (Figure 4). However, MALAT1 was positively related to macrophage, M0, M1, M2, and macrophage/monocyte in OV. These results showed that MALAT could play a crucial role in regulating macrophage in tumor.

### The correlation of MALAT1 expression with T cells

**Figure 5.**
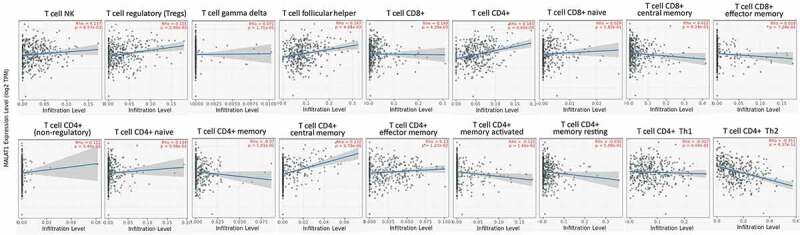
**The correlation of MALAT1 expression with T cells**. The expression of MALAT1 was significantly positively correlated with T-cell NK, Tregs, T-cell follicular helper, T-cell CD8+, T-cell CD4+, T-cell CD4+ (nonregulatory), T-cell CD4+ naïve, T-cell CD4+ central memory, and T-cell CD4+ effector memory except for T-cell CD4+ memory activated and T-cell CD4+ Th2 (negative correlation) in BLCA

T-cell mediated anti-tumor immune response exerts a key role in adaptive immunity [27,28]. Therefore, dysfunction of anti-tumor T cells may be the main reason why tumor initiated. Here, we studied the correlation of MALAT1 level with T cells in tumors. The mRNA level of MALAT1 was positively associated with T-cell NK, Tregs, T-cell follicular helper, T-cell CD8+, T-cell CD4+, T-cell CD4+ (nonregulatory), T-cell CD4+ naïve, T-cell CD4+ central memory, and T-cell CD4+ effector memory except for T-cell CD4+ memory activated and T-cell CD4+ Th2 (negative correlation) in BLCA (Figure 5). In LUAD, MALAT1 mRNA level was positively correlated with Tregs, T-cell follicular helper, T-cell CD8+, T-cell CD4+, T-cell CD8+ effector memory, T-cell CD4+ naïve, T-cell CD4+ central memory, T-cell CD4+ memory resting, and T-cell CD4+ Th1, however, negatively correlated with T-cell CD4+ (nonregulatory), T-cell CD4+ memory, T-cell CD4+ memory activated, and T-cell CD4+ Th2 (Supplementary figure 4). These data further suggested that MALAT1 might function regulating immune cell infiltrating in tumor microenvironments.

## Discussion

The length of human MALAT1 reached about 8.7 knt and mouse RNA was shorter (~6.7knt) than human RNA [29]. MALAT1 was one of earlier investigated long noncoding RNAs in tumor and other fields [8,30]. Previous studies already illustrated the vital role of MALAT1 in tumor developments including cell growth and metastasis [31–33]. The oncogenic role of MALAT1 was generally recognized in these published papers. However, the functions of MALAT1 in vitro and in vivo studies were sometimes the polar opposite.

In the terms of MALAT1 expression in tumor patients, most of the published papers showed the overexpression of MALT1 in tumor samples. Nevertheless, some studies showed the downregulation of MALA1 in some kinds of tumors even if at the same type. By qRT-PCR analysis for 124 lung cancer patients, the rate for MALAT1 RNA level with upregulaton was 88.7％, thus indicating the oncogenic roles of MALAT1 in lung cancer patients [34]. Chen et al. reported that MALAT1 mRNA was upregulated in NSCLC tissues compared with the matched normal tissues. MALAT1 RNA level correlated with the age, tumor size, and TNM stage [35]. The other studies also demonstrated the high expression of MALAT1 in NSCLC tissues or cells and the association with tumor size and lymphatic metastasis [36–38]. In situ hybridization results showed that MALAT1 was high expressed in NSCLC tissues and not detected in nonmalignant normal lung tissue. However, there was no correlation between MALAT1 level and clinical features [39]. In breast cancer, Ou et al. reported MALAT1 was upregulated in triple-negative breast cancer and associated with clinical clinicopathological parameters (tumor size, LNMET, and TNM stage) [40]. Moreover, the high level of MALAT1 in breast cancer has been demonstrated and was related to tumor stage and size in other studies [41–44]. Oncogenic function of MALAT1 in breast cancer is correspondingly found [45–47], whereas Kim et al. reported that MALAT1 suppressed breast cancer metastasis [48]. Several other studies also confirmed the inhibitory function of MALAT1 in breast cancer [49,50]. In glioma, distinct conclusion was also shown in tumor growth and metastasis [51–55]. Some researchers illustrated the aberrantly increased expression of MALAT1 in glioma tissues and cells, and the correlation with worse prognosis in glioma patients [55,56]. Cao et al demonstrated that RNA level of MALAT1 was decreased in glioma tissues compared with the noncancerous brain tissues. MALAT1 RNA level also related to tumor size, WHO grade, and Karnofsky Performance Status (KPS) [54]. Han et al. demonstrated that the RNA level of MALAT1 was lower in glioma compared with normal brain tissues [57]. In gastric cancer, MALAT1 RNA level was more than normal tissue [58–61].

In the present research, we demonstrated that MALAT1 level was upregulated only in esophageal cancer, acute myeloid leukemia, and stomach adenocarcinoma, and widely low expressed in most kinds of cancers, including lung cancer etc., by GEPIA. However, another two databases (Oncomine and TIMER) demonstrated different expression level of MALAT1 in cancers. This may be due to the differences in data collection and processing among different databases. Especially, we also found that upregulation of MALAT1 RNA level was related to early stage in lung and gastric cancer patients.

Concerning the prognostic role, there was a correlation between MALAT1 RNA level and survival of tumor patients. Generally, MALAT1 high RNA level in multiple tumor tissues is related to poor patient prognosis [62–64]. At beginning, MALAT1 was found to be a prognostic parameter for stage I NSCLC survival by Kaplan–Meier analysis [8]. High RNA level of MALAT1 contributed to a poor outcome of lung cancer patients, and Kaplan–Meier analysis showed that high RNA level of MALAT1 was related to poor overall survival in lung cancer patients [34]. NSCLC patients with upregulated MALAT1 showed worse OS rates [65–67]. Schmidt et al. reported that for squamous cell carcinoma, strong MALAT-1 RNA level was correlated with poor prognosis, whereas for nonsquamous cell carcinoma, positive MALAT-1 expression did not show a significant effect on prognosis [39]. Jadaliha et al. showed that the upregulated MALAT1 expression was associated with decreased disease-specific survival in ER negative, lymph node negative patients of the HER2, and TNBC molecular subtypes [68]. In the plasma for gastric cancer patients, MALAT1 RNA level was higher than controls and might be applied to prognosis [58]. MALAT1 is related to the poor clinical prognosis of patients with breast cancer [40,69,70]. In this study, we found that MALAT1 RNA level was related to 11 types of cancers, including BLCA, SKCM, PRAD, and so on. And MALAT1 expression had a worse or better prognosis across different cancers. Given the dual roles in cancer, we might need to precisely employ this strategy for clinical application according to the type of cancer.

Tumor microenvironment offered the tumor cells a breeding ground to grow and evolve [71–73]. Tumor microenvironment includes various kinds of immune cells, including T cells, B cells, and macrophage and so on to develop a tumor immune environment [74,75]. The tumor immune environment occupies the important role in fighting against tumor cells [76,77]. However, some immune cells also might be bribed by tumor cells to protect enemies [78–81]. It is essential to understand the function and mechanism of immune cells and the association of immune cells with some key genes. Here, MALAT1 expression was related to immune cell infiltrating in lung and bladder cancer. The RNA level of MALAT1 was significantly related to the tumor purity in eight types of tumors. In addition, there was a relation between MALAT1 RNA level and infiltrating immune cell in various tumors. In this analysis, we found that MALAT1 RNA level was significantly related to macrophage in multiple tumors (26 in 40 types of tumors). MALAT could play a crucial role in regulating macrophage in tumor. MALAT1 correlated with T-cell NK, Tregs, T-cell follicular helper, T-cell CD8+, T-cell CD4+, T-cell CD4+ (nonregulatory), T-cell CD4+ naïve, T-cell CD4+ central memory, and T-cell CD4+ effector memory except for T-cell CD4+ memory activated and T-cell CD4+ Th2 (negative correlation) in BLCA. MALAT1 might function regulating immune cell infiltrating in tumor microenvironments. Several similar works were found in recent studies however, there was no report about the comprehensive analysis of MALAT1 across various cancers by multiple databases [82,83].

## Conclusion

In conclusion, we analyzed the expression of MALAT1 and prognostic significance across different cancers from different public databases. We also identified the immunological significance of MALAT1 in various kinds of cancers and demonstrated that MALAT1 was closely correlated with the infiltrating immune cell in various tumors. Therefore, MALAT1 might play a vital role in tumor prognosis and immune regulation.

## Supplementary Material

Supplemental Material
