# Supplementary material for: Prognostic and immunological significance of metastasis associated lung adenocarcinoma transcript 1 among different kinds of cancers
Source: Bioengineered. 2021 Jul 24;12(1):4247–58. doi: 10.1080/21655979.2021.1955511 (PMC8806457; doi:10.1080/21655979.2021.1955511)
Supplement: Supplemental Material [file KBIE_A_1955511_SM6795.zip › supplementary/3 Supplementary data.docx]

**Supplementary data**

**Supplementary Figure 1** PrognoScan database was used to analyze prognostic value of MALAT1 in human cancers.

**Supplementary Figure 2** The expression of MALAT1 was significantly correlated with the tumor purity in 8 types of tumors.

**Supplementary figure 3** In BLCA, MALAT1 expression was associated with immune cell infiltration.

**Supplementary figure 4** In LUAD, MALAT1 expression was significantly positively correlated with T cell infiltration.
