# Supplementary material for: Prognostic and immunological significance of metastasis associated lung adenocarcinoma transcript 1 among different kinds of cancers
Source: Bioengineered. 2021 Jul 24;12(1):4247–58. doi: 10.1080/21655979.2021.1955511 (PMC8806457; doi:10.1080/21655979.2021.1955511)
Supplement: Supplemental Material [file KBIE_A_1955511_SM6795.zip › supplementary/4 Supplementary table 1.docx]

Supplementary table 1 | Correlation of MALAT1 expression and clinical prognosis in lung cancer by Kaplan-Meier plotter.

| Clinicopathological characteristics | Overall survival | | | First Progression | | |
| --- | --- | --- | --- | --- | --- | --- |
|  | n | Hazard ratio | *P*-value | n | Hazard ratio | *P*-value |
| Sex |  |  |  |  |  |  |
| Female | 374 | 0.54(0.38-0.77) | 0.0006 | 253 | 0.67(0.43-1.05) | 0.0815 |
| Male | 659 | 0.67(0.54-0.83) | 0.0002 | 343 | 0.65(0.46-0.91) | 0.0124 |
| Smoking history |  |  |  |  |  |  |
| exclude those never smoked | 300 | 0.53(0.34-0.8) | 0.0026 | 297 | 0.85(0.57-1.26) | 0.4127 |
| only those never smoked | 141 | 0.8(0.36-1.79) | 0.5875 | 141 | 0.68(0.36-1.27) | 0.2199 |
| Histology |  |  |  |  |  |  |
| Adenocarcinoma | 672 | 0.56(0.43-0.72) | 6.2e-6 | 443 | 0.52(0.38-0.73) | 0.0001 |
| Squamous cell carcinoma | 271 | 1.05(0.76-1.44) | 0.7797 | 141 | 0.76(0.45-1.26) | 0.2825 |
|  |  |  |  |  |  |  |
| Stage |  |  |  |  |  |  |
| 1 | 449 | 0.39(0.28-0.56) | 5.6e-8 | 316 | 0.54(0.34-0.85) | 0.0072 |
| 2 | 161 | 0.62(0.39-0.98) | 0.0406 | 125 | 1.2(0.7-2.04) | 0.5131 |
| 3 | 44 | 0.72(0.35-1.48) | 0.3702 |  |  |  |
| Stage T |  |  |  |  |  |  |
| 1 | 224 | 0.62(0.41-0.93) | 0.0204 | 54 | 0.34(0.07-1.63) | 0.1575 |
| 2 | 190 | 0.89(0.6-1.3) | 0.5385 | 121 | 1.12(0.61-2.08) | 0.7076 |
| 3 | 29 | 0.72(0.32-1.61) | 0.4247 |  |  |  |
| 4 | 23 | 0.97(0.41-2.25) | 0.9369 |  |  |  |
| Stage N |  |  |  |  |  |  |
| 0 | 324 | 0.78(0.57-1.07) | 0.1285 | 126 | 0.92(0.46-1.85) | 0.8124 |
| 1 | 102 | 0.8(0.48-1.32) | 0.3769 | 51 | 1.03(0.42-2.55) | 0.9414 |
| 2 | 32 | 1.17(0.56-2.47) | 0.6732 |  |  |  |
| Stage M |  |  |  |  |  |  |
| 0 | 462 | 0.73(0.57-0.94) | 0.0132 | 177 | 1.03(0.6-1.79) | 0.9053 |
| Chemotherapy |  |  |  |  |  |  |
| Yes | 34 | 0.3(0.09-1.03) | 0.0448 | 34 | 0.49(0.18-1.3) | 0.1439 |
| No | 21 | 0.6(0.11-3.29) | 0.553 | 21 | 0.87(0.26-2.87) | 0.8204 |
