# Supplementary figures and images for: Prognostic and immunological significance of metastasis associated lung adenocarcinoma transcript 1 among different kinds of cancers
Source: Bioengineered. 2021 Jul 24;12(1):4247–58. doi: 10.1080/21655979.2021.1955511 (PMC8806457; doi:10.1080/21655979.2021.1955511)

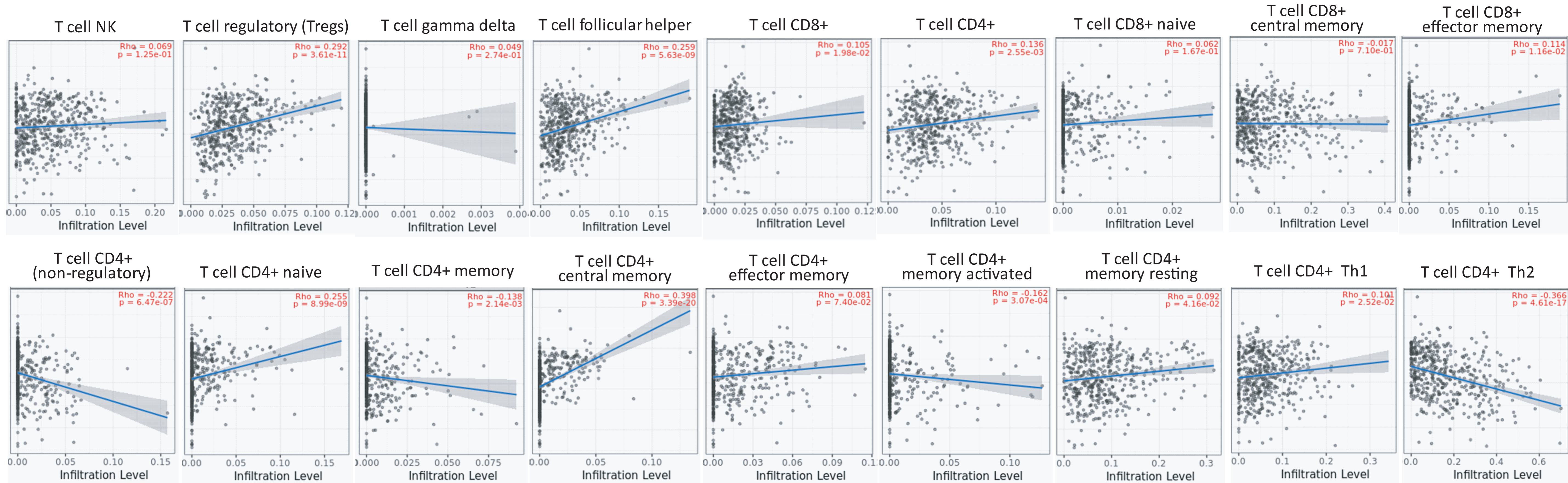

Supplement: Supplemental Material [file KBIE_A_1955511_SM6795.zip › supplementary/downloadFromZipFile.pdf]

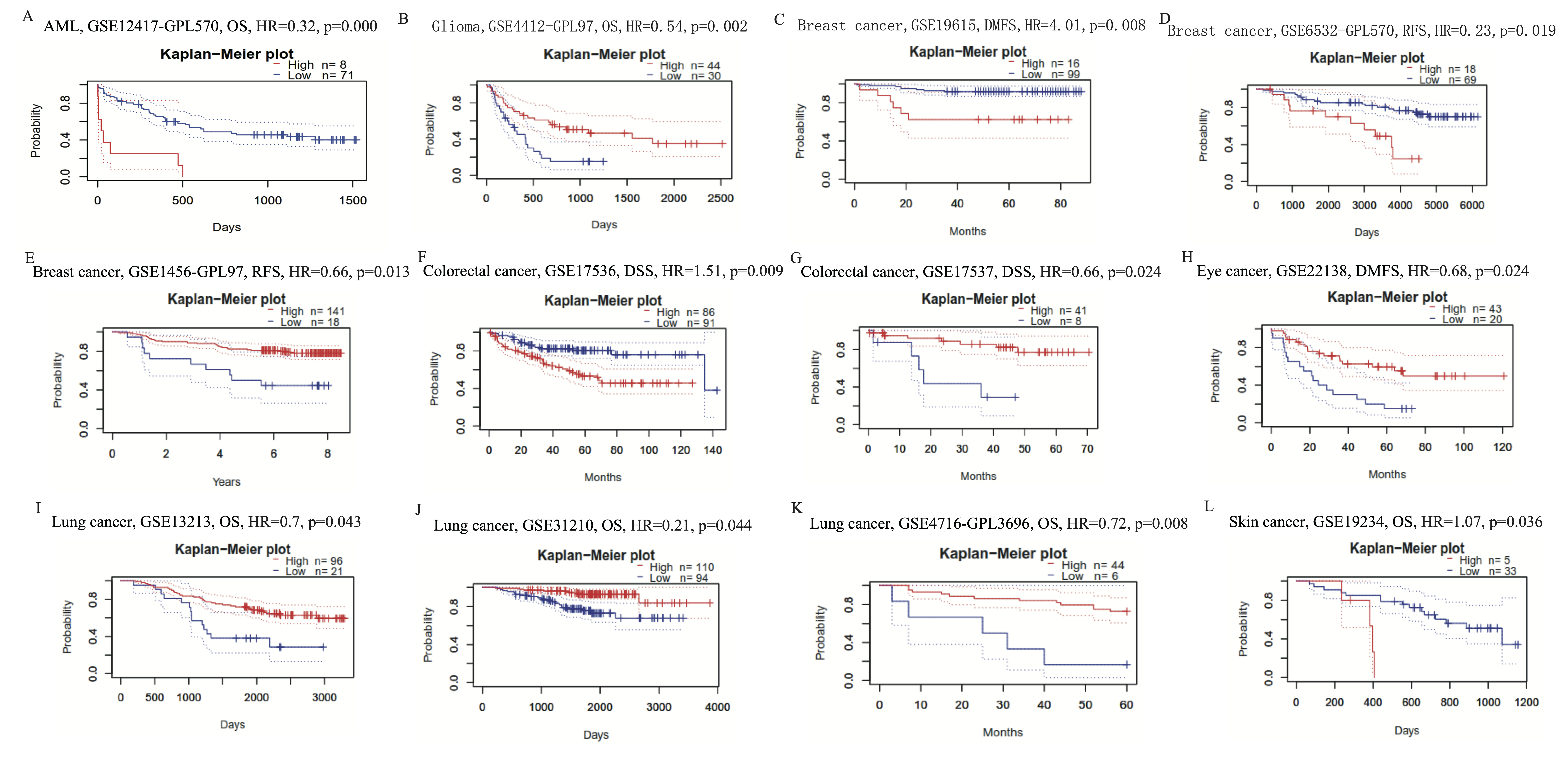

Supplement: Supplemental Material [file KBIE_A_1955511_SM6795.zip › supplementary/Supplementary_figure_1.png]

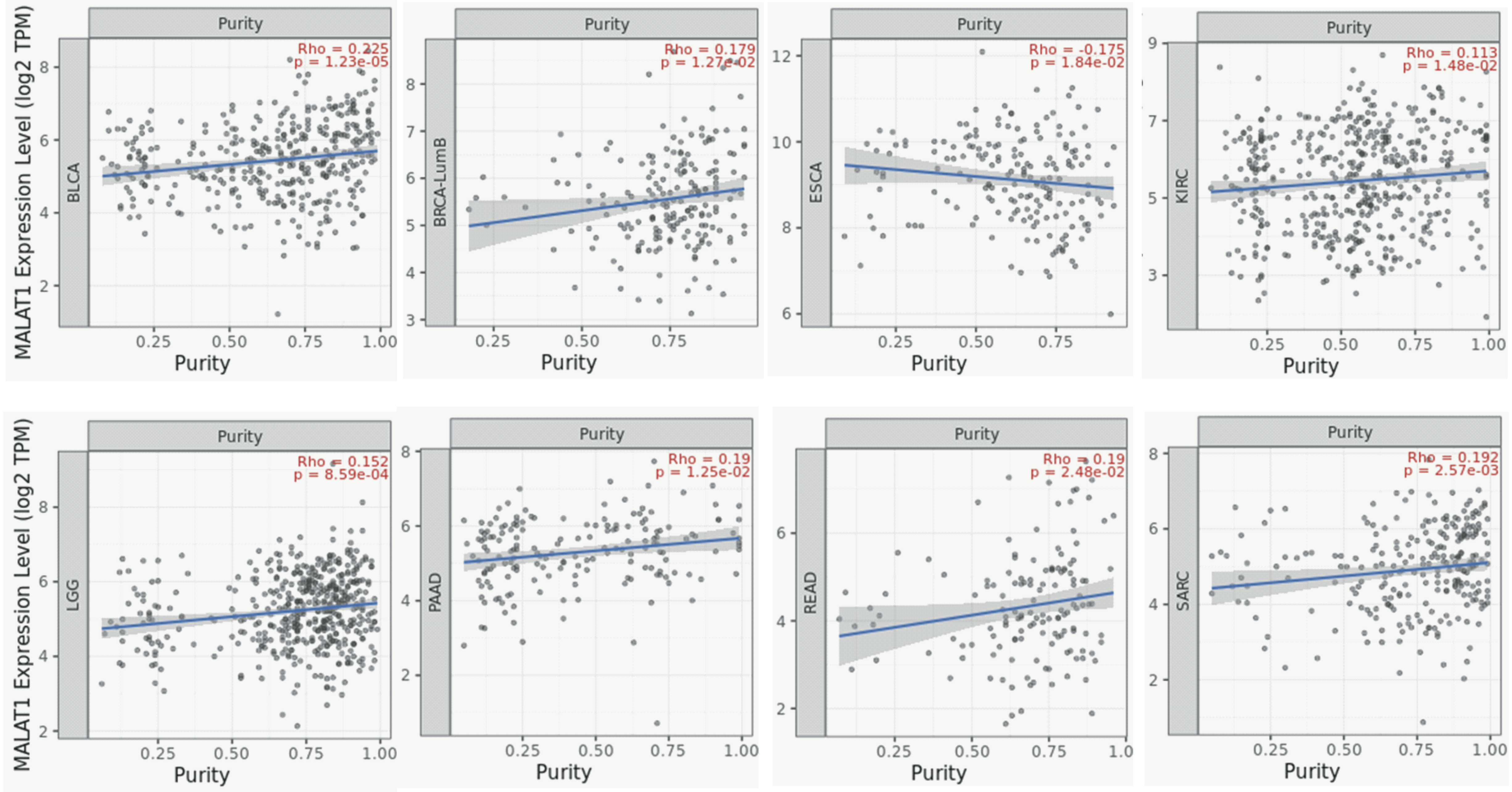

Supplement: Supplemental Material [file KBIE_A_1955511_SM6795.zip › supplementary/Supplementary_figure_2.png]

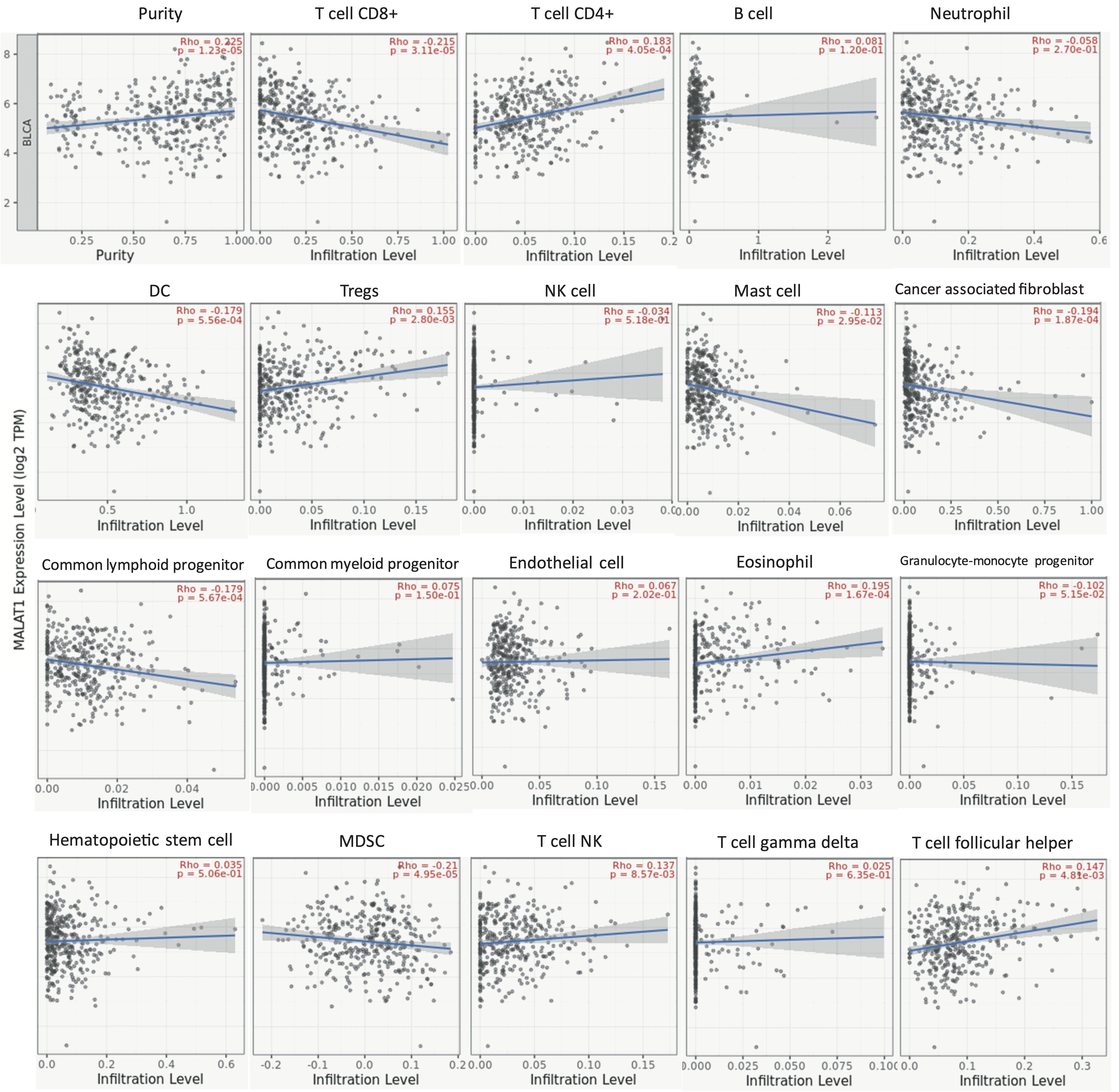

Supplement: Supplemental Material [file KBIE_A_1955511_SM6795.zip › supplementary/Supplementary_figure_3.png]
